# The Transplanting of a Rabbit’s Cornea into the Human Eye

**Published:** 1889-05

**Authors:** Julian J. Chisolm

**Affiliations:** Professor of Eye and Ear Diseases in the University of Maryland, and Surgeon-in-Chief to the Presbyterian Eye and Ear Charity Hospital of Baltimore City


### THE TRANSPLANTING OF A RABBIT'S CORNEA INTO THE HUMAN EYE.

BY JULIAN J. CHISOLM, M. D.,

*Professor of Eye and Ear Diseases in the University of Maryland, and Surgeon-in-Chief to the Presbyterian Eye and Ear Charity Hospital of Baltimore City.*

Six months since I transplanted a disk from a healthy rabbit's cornea into the cornea of a patient who had lost sight from the effects of a lime burn. For three years his eyeballs and lids had been united in symblepharon. I had liberated the left eyeball, and on two occasions had dissected away the whole anterior layer of the fleshy cornea with the hope of meeting a transparent portion within. Granulation tissue would soon form again, and a grayish red cornea would finally always remain. He had good light perception, but could not get beyond this low degree of vision. An active man, of twenty-nine years of age, he seemed doomed to permanent blindness for the remainder of his life. His constant appeals for further operations, and the desire to have me keep on cutting away the fleshy layers of granulation tissue as often as they formed, induced me to transplant a disk of clear rabbit's cornea into the centre of his opaque one.

By means of Dr. Von Hippel's ingenious instrument I succeeded in trephining the human cornea, through all of its layers, down to the membrane of Descemet. Seizing the edge of the circular-cut piece, I had the satisfaction of drawing it away entire, leaving a clear lining membrane to the anterior chamber of the eye. Through this clear peep-hole vision was at once restored. Into this well from which the trephine had taken the thick plug I inserted a disk of clear cornea cut out from a living rabbit's eye, by the same trephining instrument. The piece fitted perfectly into the hole made for it, as a bung does in the bung-hole of a barrel, its Descemet membrane lying upon the Descemet membrane of the human eye. The closed lid and a compress kept the graft in position. In a very few days the transplanted piece had become thoroughly adherent to the circular incision. From that time the rabbit's cornea has become identified with that of the man.

As to the successful transplantation of a graft there is now no longer a doubt. How the graft would behave in its new relations was the question of importance to the thousands of blind persons anxiously awaiting the final result of such experiments. To become united to the fleshy walls of the human coınea the transparent disk of rabbit's cornea had to become vascular, and necessarily opaque from infiltration. This was an essential part of the curative process, in order to establish union between the graft and its new bed, and to supply nourishment. Had it not occurred, the transferred piece must have sloughed, shrunken up, and fallen out.

It was very interesting to watch these vitalizing changes as they occurred in the graft. When the eye was exposed after five days of bandaging the infiltrated disk graft, so much whiter than the surrounding red fleshy cornea, made the new central plug quite conspicuous. Some days afterward blood vessels could be traced creeping into the graft from the pannitic margins of the human cornea, and finally the patch became quite pervaded by newly formed blood vessels. Fortunately it never became fleshy, although the individual vessels of fine calibre, looping over the circular margin at the line of adhesion, could be traced in their meanderings over the surface of the graft. They were directly connected with large feeding vessels in the ocular conjunctiva. In the course of treatment I divided these large trunks so as to cut off the excessive blood supply to the transplanted piece, and have thereby caused a visible skrinkage of the fine red lines in the graft.

After six months the new piece of transplanted cornea retains its distinguishing features, and its outlines can be readily detected. It is grayish in color, with red marginal tracings. No vessels run entirely across the disk. The back and black ground of pupil modifies decidedly the tinting of this central part of the cornea, and becomes more conspicuous monthly as the graft slowly clears up.

Now comes the question of all importance. What good has the patient secured by the experimental transplantation? Fortunately the benefits, even at this early stage of the experiment, are positive. The patient can see large objects ten feet away from him. This amount of sight, already secured, enables him to move about with confidence, when before the operation he dared not move a foot without the aid of a guiding hand. Alone and unattended he can walk for miles along the country roads in the neighborhood of his home. Quite recently he visited the hospital for inspection. Since the operation his whole facial expression is changed from one of permanent anxiety and doubt to one of confidence and cheerfulness. He can already move about with some freedom, avoiding obstacles in his way. He sees persons ten feet off but not yet to recognize them. This of itself is an immense improvement on no sight. Still I see much better things in store for him. From month to month I can mark his improvement in detecting objects which he did not see on a former visit.

This slow progress is perfectly in keeping with the pathology of the cornea. We are accustomed to observe the snail-like progress in cases of interstitial keratitis, and how months always, and even years are required to restore in such cases good sight to blind people. This piece of transplanted cornea is now undergoing the familiar and regular metamorphoses of interstitial keratitis. While the fleshy cicatricial tissue of the cornea can undergo no change for the better, and must ever remain opaque, there is every evidence that the transplanted piece, infiltrated as it still is, will in time give him the peep hole desired, and will restore useful vision to one otherwise doomed to permanent blindness.

The transplanting of a disk of rabbit's cornea for the purpose of rendering the central portion of an opaque human cornea useful, is in this case already so much of a success as to warrant its repetition on any suitable case which may present itself. All cases of corneal blindness are not suitable cases for transplanting. Most of such diseased eyes are the sequel of perforating or sloughing ulcers of the cornea, with amalgamation of iris and cicatricial tissue, and with no aqueous chamber. The *sine qua non* of a proper case for operation is a perfect elastic membrane of Descemet, and a normal anterior chamber, filled with aqueous fluid. The lens and iris must also be intact, with a free pupil. For successful transplanting there must be a transparent membrane of Descemet as the foundation upon which to plant the graft. In preparing the eye by trephining, this membrane must not be perforated. It must remain perfect so as to retain the aqueous secretion within its proper chamber, and make a barrier, to prevent the displacement of the newly placed corneal disk by oozing away of the eye humors.

In the anatomy of the cornea the various layers of which it is composed are much more closely adherent to each other than is the innermost layer of corneal tissue to the elastic membrane of Descemet. In deep ulcerations of the cornea, this lining membrane of the anterior eye chamber will so draw upon its loose connections as to form a protruding hernia through the bottom of the ulcer. By the dexterous handling of Dr. Von Hippel's trephining instrument, layer by layer of corneal tissue can be cut through without disturbing the lining membrane. By watching the depth and the regularity of the trephining section, as is necessary in opening the skull, the cupping or turning up of the edges of the portion, as freed, will indicate surely when all the layers are cut through. Seizing the loosened plug with a forceps it is much more easily removed as a whole by traction, than brought away piecemeal. Practice on the fresh eyes of slaughtered animals will make the manipulation perfect. The rabbit's cornea is not so thick as the human, but as the whole thickness of it is added to the human Descemet's membrane, this addition with the slight pushing out of the membrane by the watery contents of the chamber, will bring the surface of the graft on a level with the anterior corneal surface, and insure the nice adaptation of the edges. The two serous layers when juxtaposed one on the top of the other adhere promptly and perfectly.

In suitable cases the transplanting of a disk of clear animal cornea into the centre of an opaque human cornea, is a legitimate surgical operation, promising to give useful vision to blind people. By useful vision I do not mean perfect sight. If sufficient vision is obtained to allow one to guide himself, and to move about in safety without the constant presence of a helper, it is well worthy of an effort to secure. Judging by my experience in watching the slow development of returning sight in my patient, I would say that the little, but steady, progress in seeing from week to week will, in the course of time, re-establish an amount of vision very enviable to those who have it not. Independence against compulsory dependence in any of the affairs of life is well worth striving for. Useful sight against no sight becomes a precious acquisition. In a certain class of blind persons the transplanting of clear corneal grafts seems to be the only way to attain this this much desired and longed for blessing.—*International Journal of Surgery.*

